# Supplementary material for: On the mechanisms of brain blood flow regulation during hypoxia
Source: J Physiol. Author manuscript; Available in PMC 2025 Apr 23. (PMC12013793; doi:10.1113/JP285060)
Supplement: Table S1-S5 [file EMS204615-supplement-Table_S1_S5.pdf]

Supplementary Table 1| Search terms used in the database Medline via Ovid 1946- 17 January 2024

1	hypoxia/ or fetal hypoxia/ or hypoxia, brain/
2	hypoxi*.tw,kw.
3	oxygen deficienc*.tw,kw.
4	hypoxemia.tw,kw.
5	anoxi*.tw,kw.
6	anoxemia.tw,kw.
7	decreased oxygen tension.tw,kw.
8	1 or 2 or 3 or 4 or 5 or 6 or 7
9	Cerebrovascular Circulation/
10	Vasodilation/
11	Vasodilation.tw,kw.
12	Cerebral microvascular dilation.tw,kw.
13	Cerebral blood flow*.tw,kw.
14	Cerebrovascular Circulation.tw,kw.
15	Cerebral circulation*.tw,kw.
16	brain blood flow*.tw,kw.
17	cerebral perfusion pressure*.tw,kw.
18	cerebrovasodilation.tw,kw.
19	brain bf.tw,kw.
20	pial artery dilation.tw,kw.
21	hypoxic dilatatory arteriolar.tw,kw.
22	cerebral vasodilation.tw,kw.
23	blood flow-metabolism couple.tw,kw.
24	artery dilation.tw,kw.
25	cerebral artery velocity.tw,kw.
26	middle cerebral artery velocity.tw,kw.
27	hypoxic dilation.tw,kw.
28	pial arteriolar dilation.tw,kw.
29	pial arteriolar response.tw,kw.
30	anoxia-induced hyperemia.tw,kw.
31	cerebrovasodilation.tw,kw.
32	9 or 10 or 11 or 12 or 13 or 14 or 15 or 16 or 17 or 18 or 19 or 20 or 21 or 22 or 23 or 24 or 25 or 26 or 27 or 28 or 29 or 30 or 31
33	8 and 32
34	Adenosine.tw,kw.
35	Adenosine/
36	Theophylline/
37	Theophylline.tw,kw.
38	8-phenyltheophylline.tw,kw.
39	Adenosine Deaminase/
40	Adenosine Deaminase.tw,kw.
41	34 or 35 or 36 or 37 or 38 or 39 or 40
42	33 and 41
43	arachidonic acids/ or arachidonic acid/ or hydroxyeicosatetraenoic acids/ or isoprostanes/ or leukotrienes/ or thromboxanes/
44	arachidonic acid*.tw,kw.
45	hydroxyeicosatetraenoic acid*.tw,kw.
46	isoprostane*.tw,kw.
47	leukotriene*.tw,kw.
48	thromboxane*.tw,kw.
49	43 or 44 or 45 or 46 or 47 or 48
50	33 and 49
51	KATP Channels/
52	KATP Channel*.tw,kw.
53	ATP-sensitive K+ Channel*.tw,kw.
54	Glibenclamide.tw,kw.
55	ATP-sensitive potassium Channel*.tw,kw.
56	51 or 52 or 53 or 54 or 55
57	33 and 56
58	Cyclic GMP/
59	Cyclic GMP.tw,kw.
60	cGMP.tw,kw.
61	cyclic guanosine monophosphate*.tw,kw.
62	Methylene Blue/
63	Methylene Blue.tw,kw.
64	58 or 59 or 60 or 61 or 62 or 63
65	33 and 64
66	prostaglandin-endoperoxide synthases/ or cyclooxygenase 1/ or cyclooxygenase 2/
67	prostaglandin-endoperoxide synthase*.tw,kw.
68	cyclooxygenase 1.tw,kw.
69	cyclooxygenase 2.tw,kw.
70	Indomethacin/
71	Indomethacin.tw,kw.
72	endothelin*.tw,kw.
73	66 or 67 or 68 or 69 or 70 or 71 or 72
74	33 and 73
75	potassium channels, calcium-activated/ or intermediate-conductance calcium-activated potassium channels/ or large-conductance calcium-activated potassium channels/ or small-conductance calcium-activated potassium channels/
76	calcium-activated potassium channel*.tw,kw.
77	intermediate-conductance calcium-activated potassium channel*.tw,kw.
78	large-conductance calcium-activated potassium channel*.tw,kw.
79	small-conductance calcium-activated potassium channel*.tw,kw.
80	K* channel*.tw,kw.
81	75 or 76 or 77 or 78 or 79 or 80
82	33 and 81
83	nitric oxide synthase/ or nitric oxide synthase type i/ or nitric oxide synthase type ii/ or nitric oxide synthase type iii/
84	eNOS.tw,kw.
85	nNOS.tw,kw.
86	iNOS.tw,kw.
87	endothelial nitric oxide synthase.tw,kw.
88	neuronal nitric oxide synthase.tw,kw.
89	inducible nitric oxide synthase.tw,kw.
90	endothelial NOS.tw,kw.
91	neuronal NOS.tw,kw.
92	inducible NOS.tw,kw.
93	Nitric Oxide/
94	Nitric Oxide.tw,kw.
95	83 or 84 or 85 or 86 or 87 or 88 or 89 or 90 or 91 or 92 or 93 or 94
96	33 and 95
97	sodium channels/ or voltage-gated sodium channels/
98	sodium channel*.tw,kw.
99	voltage-gated sodium channel*.tw,kw.
100	Tetrodotoxin/
101	97 or 98 or 99 or 100
102	33 and 101
103	Epinephrine/
104	Epinephrine.tw,kw.
105	Adrenaline.tw,kw.
106	receptors, adrenergic/ or receptors, adrenergic, alpha/ or receptors, adrenergic, beta/
107	alpha adrenergic receptor*.tw,kw.
108	beta adrenergic receptor*.tw,kw.
109	adrenoreceptor*.tw,kw.
110	alpha 2-adrenoreceptor*.tw,kw.
111	alpha 1-adrenoreceptor*.tw,kw.
112	beta 2-adrenoreceptor*.tw,kw.
113	beta 1-adrenoreceptor*.tw,kw.
114	beta 3-adrenoreceptor*.tw,kw.
115	adrenergic alpha-antagonists/ or adrenergic beta-antagonists/ or adrenergic beta-1 receptor antagonists/ or adrenergic beta-2 receptor antagonists/ or adrenergic beta-3 receptor antagonists/
116	beta adrenergic antagonist*.tw,kw.
117	beta-2 adrenergic receptor antagonist*.tw,kw.
118	beta-1 adrenergic receptor antagonist*.tw,kw.
119	beta-3 adrenergic receptor antagonist*.tw,kw.
120	alpha-2 adrenergic receptor antagonist*.tw,kw.
121	alpha-1 adrenergic receptor antagonist*.tw,kw.
122	103 or 104 or 105 or 106 or 107 or 108 or 109 or 110 or 111 or 112 or 113 or 114 or 115 or 116 or 117 or 118 or 119 or 120 or 121
123	33 and 122
124	Glutamates/
125	Glutamate*.tw,kw.
126	alpha-Amino-3-hydroxy-5-methyl-4-isoxazolepropionic Acid/
127	N-Methylaspartate/
128	NMDA*.tw,kw.
129	AMPA*.tw,kw.
130	NMDAR*.tw,kw.
131	AMPAR*.tw,kw.
132	receptors, ionotropic glutamate/ or receptors, ampa/ or receptors, kainic acid/ or receptors, n-methyl-d-aspartate/
133	AMPA Receptor*.tw,kw.
134	NMDA Receptor*.tw,kw.
135	124 or 125 or 126 or 127 or 128 or 129 or 130 or 131 or 132 or 133 or 134
136	33 and 135
137	Hydrogen Sulfide/
138	Hydrogen Sulfide.tw,kw.
139	"heme oxygenase (decyclizing)"/ or heme oxygenase-1/
140	heme oxygenase.tw,kw.
141	heme oxygenase-1.tw,kw.
142	137 or 138 or 139 or 140 or 141
143	33 and 142
144	Eicosapentaenoic Acid/
145	eicosapentaenoic acid?.tw,kw.
146	5,8,11,14-eicosatetraynoic acid?.tw,kw.
147	8,11,14-eicosatrienoic acid?.tw,kw.
148	144 or 145 or 146 or 147
149	33 and 148
150	histamine/ or methylhistamines/
151	histamine*.tw,kw.
152	methylhistamine*.tw,kw.
153	receptors, histamine/ or receptors, histamine h1/ or receptors, histamine h2/ or receptors, histamine h3/ or receptors, histamine h4/
154	histamine receptor*.tw,kw.
155	histamine h1 receptor*.tw,kw.
156	histamine h2 receptor*.tw,kw.
157	histamine h3 receptor*.tw,kw.
158	histamine h4 receptor*.tw,kw.
159	150 or 151 or 152 or 153 or 154 or 155 or 156 or 157 or 158
160	33 and 159
161	42 or 50 or 57 or 65 or 74 or 82 or 96 or 102 or 123 or 136 or 143 or 149 or 160
162	indomet?acin.tw,kw.
163	73 or 162
164	33 and 163
165	42 or 50 or 57 or 65 or 82 or 102 or 123 or 136 or 143 or 149 or 160 or 164
166	96 or 165
167	cyclooxygenase*.tw,kw.
168	66 or 67 or 68 or 69 or 70 or 71 or 72 or 167
169	33 and 168
170	42 or 50 or 57 or 65 or 82 or 96 or 102 or 123 or 136 or 143 or 149 or 160 or 169

Supplementary Table 2| Search terms used in the database Embase + Embase classic via Ovid 1947- 17 January 2024

1	hypoxi*.tw,kw.
2	oxygen deficienc*.tw,kw.
3	hypoxemia.tw,kw.
4	anoxi*.tw,kw.
5	anoxemia.tw,kw.
6	decreased oxygen tension.tw,kw.
7	hypoxia/ or brain hypoxia/ or newborn hypoxia/
8	1 or 2 or 3 or 4 or 5 or 6 or 7
9	Vasodilation.tw,kw.
10	Cerebral microvascular dilation.tw,kw.
11	Cerebral blood flow*.tw,kw.
12	Cerebrovascular Circulation.tw,kw.
13	Cerebral circulation*.tw,kw.
14	brain blood flow*.tw,kw.
15	cerebral perfusion pressure*.tw,kw.
16	cerebrovasodilation.tw,kw.
17	brain bf.tw,kw.
18	pial artery dilation.tw,kw.
19	hypoxic dilatatory arteriolar.tw,kw.
20	cerebral vasodilation.tw,kw.
21	blood flow-metabolism couple.tw,kw.
22	artery dilation.tw,kw.
23	cerebral artery velocity.tw,kw.
24	middle cerebral artery velocity.tw,kw.
25	hypoxic dilation.tw,kw.
26	pial arteriolar dilation.tw,kw.
27	pial arteriolar response.tw,kw.
28	anoxia-induced hyperemia.tw,kw.
29	cerebrovasodilation.tw,kw.
30	9 or 10 or 11 or 12 or 13 or 14 or 15 or 16 or 17 or 18 or 19 or 20 or 21 or 22 or 23 or 24 or 25 or 26 or 27 or 28 or 29
31	8 and 30
32	Adenosine.tw,kw.
33	8-phenyltheophylline.tw,kw.
34	Theophylline.tw,kw.
35	Adenosine Deaminase.tw,kw.
36	Adenosine Deaminase/
37	Theophylline/
38	Adenosine/
39	32 or 33 or 34 or 35 or 36 or 37 or 38
40	31 and 39
41	arachidonic acid*.tw,kw.
42	hydroxyeicosatetraenoic acid*.tw,kw.
43	isoprostane*.tw,kw.
44	leukotriene*.tw,kw.
45	thromboxane*.tw,kw.
46	arachidonic acid/
47	41 or 42 or 43 or 44 or 45 or 46
48	31 and 47
49	KATP Channel*.tw,kw.
50	ATP-sensitive K+ Channel*.tw,kw.
51	Glibenclamide.tw,kw.
52	ATP-sensitive potassium Channel*.tw,kw.
53	adenosine triphosphate sensitive potassium channel/ or inwardly rectifying potassium channel/
54	49 or 50 or 51 or 52 or 53
55	31 and 54
56	Cyclic GMP.tw,kw.
57	cGMP.tw,kw.
58	cyclic guanosine monophosphate*.tw,kw.
59	Methylene Blue/
60	Methylene Blue.tw,kw.
61	cyclic GMP/
62	56 or 57 or 58 or 59 or 60 or 61
63	31 and 62
64	prostaglandin-endoperoxide synthase*.tw,kw.
65	cyclooxygenase 1.tw,kw.
66	cyclooxygenase 2.tw,kw.
67	Indomethacin.tw,kw.
68	endothelin*.tw,kw.
69	cyclooxygenase.tw,kw.
70	prostaglandin synthase/
71	64 or 65 or 66 or 67 or 68 or 69 or 70
72	31 and 71
73	calcium-activated potassium channel*.tw,kw.
74	intermediate-conductance calcium-activated potassium channel*.tw,kw.
75	large-conductance calcium-activated potassium channel*.tw,kw.
76	small-conductance calcium-activated potassium channel*.tw,kw.
77	K* channel*.tw,kw.
78	calcium activated potassium channel/ or intermediate conductance calcium activated potassium channel/ or large conductance calcium activated potassium channel/ or small conductance calcium activated potassium channel/
79	73 or 74 or 75 or 76 or 77 or 78
80	31 and 79
81	eNOS.tw,kw.
82	nNOS.tw,kw.
83	iNOS.tw,kw.
84	endothelial nitric oxide synthase.tw,kw.
85	neuronal nitric oxide synthase.tw,kw.
86	inducible nitric oxide synthase.tw,kw.
87	endothelial NOS.tw,kw.
88	neuronal NOS.tw,kw.
89	inducible NOS.tw,kw.
90	Nitric Oxide.tw,kw.
91	nitric oxide/
92	nitric oxide synthase/
93	81 or 82 or 83 or 84 or 85 or 86 or 87 or 88 or 89 or 90 or 91 or 92
94	31 and 93
95	sodium channel/ or voltage gated sodium channel/
96	sodium channel*.tw,kw.
97	voltage-gated sodium channel*.tw,kw.
98	tetrodotoxin/
99	tetrodotoxin.tw,kw.
100	95 or 96 or 97 or 98 or 99
101	31 and 100
102	Epinephrine.tw,kw.
103	Adrenaline.tw,kw.
104	epinephrine/
105	alpha adrenergic receptor*.tw,kw.
106	beta adrenergic receptor*.tw,kw.
107	adrenoreceptor*.tw,kw.
108	alpha 2-adrenoreceptor*.tw,kw.
109	alpha 1-adrenoreceptor*.tw,kw.
110	beta 2-adrenoreceptor*.tw,kw.
111	beta 1-adrenoreceptor*.tw,kw.
112	beta 3-adrenoreceptor*.tw,kw.
113	beta adrenergic antagonist*.tw,kw.
114	beta-2 adrenergic receptor antagonist*.tw,kw.
115	beta-1 adrenergic receptor antagonist*.tw,kw.
116	beta-3 adrenergic receptor antagonist*.tw,kw.
117	alpha-2 adrenergic receptor antagonist*.tw,kw.
118	alpha-1 adrenergic receptor antagonist*.tw,kw.
119	adrenergic receptor blocking agent/ or alpha adrenergic receptor blocking agent/ or beta adrenergic receptor blocking agent/
120	adrenergic receptor/ or alpha adrenergic receptor/ or beta adrenergic receptor/
121	102 or 103 or 104 or 105 or 106 or 107 or 108 or 109 or 110 or 111 or 112 or 113 or 114 or 115 or 116 or 117 or 118 or 119 or 120
122	31 and 121
123	Glutamate*.tw,kw.
124	NMDA*.tw,kw.
125	AMPA*.tw,kw.
126	NMDAR*.tw,kw.
127	AMPAR*.tw,kw.
128	AMPA Receptor*.tw,kw.
129	NMDA Receptor*.tw,kw.
130	glutamic acid derivative/
131	alpha amino 3 hydroxy 5 methyl 4 isoxazolepropionic acid/
132	n methyl dextro aspartic acid/
133	glutamate receptor/
134	123 or 124 or 125 or 126 or 127 or 128 or 129 or 130 or 131 or 132 or 133
135	31 and 134
136	Hydrogen Sulfide.tw,kw.
137	heme oxygenase.tw,kw.
138	heme oxygenase-1.tw,kw.
139	heme oxygenase 1/ or heme oxygenase/
140	hydrogen sulfide/
141	136 or 137 or 138 or 139 or 140
142	31 and 141
143	eicosapentaenoic acid*.tw,kw.
144	5,8,11,14-eicosatetraynoic acid*.tw,kw.
145	8,11,14-eicosatrienoic acid*.tw,kw.
146	icosapentaenoic acid/
147	143 or 144 or 145 or 146
148	31 and 147
149	histamine*.tw,kw.
150	methylhistamine*.tw,kw.
151	histamine receptor*.tw,kw.
152	histamine h1 receptor*.tw,kw.
153	histamine h2 receptor*.tw,kw.
154	histamine h3 receptor*.tw,kw.
155	histamine h4 receptor*.tw,kw.
156	histamine receptor/ or histamine h1 receptor/ or histamine h2 receptor/ or histamine h3 receptor/ or histamine h4 receptor/
157	histamine/
158	149 or 150 or 151 or 152 or 153 or 154 or 155 or 156 or 157
159	31 and 158
160	40 or 48 or 55 or 63 or 72 or 80 or 94 or 101 or 122 or 135 or 142 or 148 or 159
161	Vasodilatation/
162	Brain circulation/
163	Brain blood flow/
164	30 or 161 or 162 or 163
165	8 and 164
166	8 phenyltheophylline/
167	32 or 33 or 34 or 35 or 36 or 37 or 38 or 166
168	Thromboxane/
169	Leukotriene/
170	hydroxyicosatetraenoic acid/
171	47 or 168 or 169 or 170
172	165 and 167
173	165 and 171
174	glibenclamide/
175	54 or 174
176	cyclooxygenase 2/
177	endothelin/
178	indomethacin/
179	cyclooxygenase 1/
180	cyclooxygenase*.tw,kw.
181	71 or 176 or 177 or 178 or 179 or 180
182	165 and 181
183	165 and 175
184	158 and 165
185	147 and 165
186	142 and 165
187	135 and 165
188	122 and 165
189	100 and 165
190	93 and 165
191	172 or 173 or 182 or 183 or 184 or 185 or 186 or 187 or 188 or 189 or 190

Supplementary Table 3| Search terms used in the database CINAHL Plus via EBSCOhost 1947- 17 January 2024

S58	S14 OR S18 OR S20 OR S24 OR S28 OR S30 OR S34 OR S36 OR S41 OR S45 OR S47 OR S49 OR S53 OR S57
S57	S12 AND S56
S56	S54 OR S55
S55	TI (histamine* OR methylhistamine* OR "histamine receptor*" OR " histamine h1 receptor*" OR " histamine h2 receptor*" OR " histamine h3 receptor*" OR "histamine h4 receptor*") OR AB (histamine* OR methylhistamine* OR "histamine receptor*" OR " histamine h1 receptor*" OR " histamine h2 receptor*" OR " histamine h3 receptor*" OR "histamine h4 receptor*")
S54	(MH "Histamine") OR (MH "Histamine H2 Antagonists") OR (MH "Histamine H1 Antagonists") OR (MH "Histamine Antagonists")
S53	S12 AND S52
S52	S50 OR S51
S51	TI ("eicosapentaenoic acid*" OR "5,8,11,14-eicosatetraynoic acid*" OR "8,11,14-eicosatrienoic acid*") OR AB ("eicosapentaenoic acid*" OR "5,8,11,14-eicosatetraynoic acid*" OR "8,11,14-eicosatrienoic acid*")
S50	(MH "Eicosapentaenoic Acid")
S49	S12 AND S48
S48	TI ("heme oxygenase" OR "heme oxygenase-1") OR AB ("heme oxygenase" OR "heme oxygenase-1")
S47	S12 AND S46
S46	S43 OR S44
S45	S12 AND S42
S44	TI ("hydrogen sulfide" or h2s) OR AB ("hydrogen sulfide" or h2s)
S43	(MH "Hydrogen Sulfide")
S42	TI ("Glutamate*" OR "alpha-Amino-3-hydroxy-5-methyl-4-isoxazolepropionic Acid" OR "NMDA*" OR "AMPA*" OR "NMDAR*" OR "AMPAR*" OR "AMPA Receptor*" OR "NMDA Receptor*" OR "ionotropic glutamate" OR "kainic acid receptor*") OR AB ("Glutamate*" OR "alpha-Amino-3-hydroxy-5-methyl-4-isoxazolepropionic Acid" OR "NMDA*" OR "AMPA*" OR "NMDAR*" OR "AMPAR*" OR "AMPA Receptor*" OR "NMDA Receptor*" OR "ionotropic glutamate" OR "kainic acid receptor*")
S41	S12 AND S40
S40	S37 OR S38 OR S39
S39	(MH "Receptors, Adrenergic") OR (MH "Receptors, Adrenergic, Alpha")
S38	TI (Epinephrine OR Adrenaline OR "alpha adrenergic receptor*" OR "beta adrenergic receptor*" OR adrenoreceptor* OR "alpha 2-adrenoreceptor*" OR "alpha 1-adrenoreceptor*" OR "beta 2-adrenoreceptor*" OR "beta 1-adrenoreceptor*" OR "beta 3-adrenoreceptor*" OR "beta adrenergic antagonist" OR "beta-2 adrenergic receptor antagonist*" OR "beta-1 adrenergic receptor antagonist*" OR "beta-3 adrenergic receptor antagonist*" OR "alpha-2 adrenergic receptor antagonist*" OR "alpha-1 adrenergic receptor antagonist*") OR AB (Epinephrine OR Adrenaline OR "alpha adrenergic receptor*" OR "beta adrenergic receptor*" OR adrenoreceptor* OR "alpha 2-adrenoreceptor*" OR "alpha 1-adrenoreceptor*" OR "beta 2-adrenoreceptor*" OR "beta 1-adrenoreceptor*" OR "beta 3-adrenoreceptor*" OR "beta adrenergic antagonist" OR "beta-2 adrenergic receptor antagonist*" OR "beta-1 adrenergic receptor antagonist*" OR "beta-3 adrenergic receptor antagonist*" OR "alpha-2 adrenergic receptor antagonist*" OR "alpha-1 adrenergic receptor antagonist*")
S37	(MH "Epinephrine")
S36	S12 AND S35
S35	TI ("sodium channel*" OR " voltage-gated sodium channel*" OR Tetrodotoxin) OR AB ("sodium channel*" OR " voltage-gated sodium channel*" OR Tetrodotoxin) OR AB ("sodium channel*" OR " voltage-gated sodium channel*" OR Tetrodotoxin) OR AB ("sodium channel*" OR " voltage-gated sodium channel*" OR Tetrodotoxin)
S34	S12 AND S33
S33	S31 OR S32
S32	TI ("Nitric Oxide Synthase*" OR "nitric oxide synthase type i" OR "nitric oxide synthase type ii" OR "nitric oxide synthase type iii" OR "eNOS" OR "nNOS" OR "iNOS" OR "endothelial nitric oxide synthase" OR "neuronal nitric oxide synthase" OR " inducible nitric oxide synthase" OR "endothelial NOS" OR "neuronal NOS" OR " inducible NOS" OR
S31	(MH "Nitric Oxide Synthases") OR (MH "Nitric Oxide")
S30	S12 AND S29
S29	TI ("calcium-activated potassium channel*" OR "intermediate-conductance calcium-activated potassium channel*" OR "large-conductance calcium-activated potassium channel*" OR "small-conductance calcium-activated potassium channel*" OR "K* channel*") OR AB ("calcium-activated potassium channel*" OR
S28	S12 AND S27
S27	S25 OR S26
S26	TI ("prostaglandin-endoperoxide synthase*" OR "cyclooxygenase 1" OR "cyclooxygenase 2" OR "Indomethacin" OR "endothelin*" OR "Prostaglandins I" OR "Prostaglandins E" OR "Prostaglandins F, Synthetic") OR AB ("prostaglandin-endoperoxide synthase*" OR "cyclooxygenase 1" OR "cyclooxygenase 2" OR "Indomethacin" OR "endothelin*" OR "Prostaglandins I" OR "Prostaglandins E" OR "Prostaglandins F, Synthetic")
S25	(MH "Prostaglandins I") OR (MH "Prostaglandins E") OR (MH "Prostaglandins F, Synthetic") OR (MH "Endothelins") OR (MH "Thromboxanes")
S24	S12 AND S23
S23	S21 OR S22
S22	(MH "Methylene Blue")
S21	("Cyclic GMP" OR cGMP OR "cyclic guanosine monophosphate" OR "Methylene Blue") OR ("Cyclic GMP" OR cGMP OR "cyclic guanosine monophosphate" OR "Methylene Blue")
S20	S12 AND S19
S19	TI ("KATP Channel*" OR "ATP-sensitive K+ Channel*" OR "Glibenclamide" OR "ATP-sensitive potassium Channel*") OR AB ("KATP Channel*" OR "ATP-sensitive K+ Channel*" OR "Glibenclamide" OR "ATP-sensitive potassium Channel*")
S18	S12 AND S17
S17	S15 OR S16
S16	TI ("arachidonic acid*" OR "hydroxyeicosatetraenoic acid*" OR "isoprostane*" OR "leukotriene*" OR "thromboxane*") OR AB ("arachidonic acid*" OR "hydroxyeicosatetraenoic acid*" OR "isoprostane*" OR "leukotriene*" OR "thromboxane*")
S15	(MH "Arachidonic Acids")
S14	S12 AND S13
S13	S8 OR S9 OR S10 OR S12
S12	S4 AND S11
S11	S5 OR S6 OR S7
S10	(Adenosine OR Theophylline OR "8-phenyltheophylline" OR "Adenosine Deaminase") OR (Adenosine OR Theophylline OR "8-phenyltheophylline" OR "Adenosine Deaminase")
S9	(MH "Theophylline")
S8	(MH "Adenosine")
S7	TI (Vasodilation OR "Cerebrovascular Circulation" OR "Cerebral blood flow*" OR "Cerebrovascular Circulation" OR "Cerebral circulation*" OR "brain blood flow*" OR "cerebral perfusion pressure*" OR "cerebrovasodilation" OR " brain bf" OR "pial artery dilation" OR "hypoxic dilatatory arteriolar" OR "blood flow-metabolism couple" OR "artery dilation" OR "cerebral artery velocity" OR "middle cerebral artery velocity" OR "hypoxic dilation" OR "pial arteriolar dilation" OR "pial arteriolar response" OR "anoxia-induced hyperemia" OR "cerebrovasodilation") OR AB (Vasodilation OR "Cerebrovascular Circulation" OR "Cerebral blood flow*" OR "Cerebrovascular Circulation" OR "Cerebral circulation*" OR "brain blood flow*" OR "cerebral perfusion pressure*" OR "cerebrovasodilation" OR " brain bf" OR "pial artery dilation" OR "hypoxic dilatatory arteriolar" OR "blood flow-metabolism couple" OR "artery dilation" OR "cerebral artery velocity" OR "middle cerebral artery velocity" OR "hypoxic dilation" OR "pial arteriolar dilation" OR "pial arteriolar response" OR "anoxia-induced hyperemia" OR "cerebrovasodilation")
S6	(MH "Vasodilation")
S5	(MH "Cerebrovascular Circulation")
S4	S1 OR S2 OR S3
S3	(MH "Anoxia")
S2	(MH "Hypoxia, Brain")
S1	TI (hypoxi* OR "oxygen deficienc*" OR hypoxemia OR anoxi* OR anoxemia OR "decreased oxygen tension") OR AB (hypoxi* OR "oxygen deficienc*" OR hypoxemia OR anoxi* OR anoxemia OR "decreased oxygen tension")

Supplementary Table 4| Characteristics of selected human studies.

AA, arachidonic acid; BMI, body mass index; FiO_2, fraction of inspired oxygen; K_{ATP} channels, ATP-sensitive potassium channels; L-NMMA, N(G)-mehtyl-L-arginine; SaO_2, arterial oxygen saturation; SpO_2 peripheral oxygen saturation.

Publication	Pathway	Age (years)	Weight or BMI	Sex	Technique	Method of induction of hypoxia	Intervention	Route of administration	N number
Bowton 1988	Adenosine	35 ± 4.5	Not stated	Male	^{133}Xe washout	Inspired O_2 reduced to achieve SaO_2 of 80%	Aminophylline (6 mg/kg)	I.V.	5
Fan 2011	AA derivatives	30 ± 10	23 ± 2 kg/m²	Both	Transcranial doppler ultrasound	Use of a rebreathing circuit to achieve an SpO_2 of 80%	Indomethacin (100 mg)	Oral	12
Hoiland 2017	Adenosine	26 ± 5.6	24 ± 3 kg/m²	Both	Duplex ultrasound	End-tidal forcing to achieve SaO_2 of 80%	Theophylline (3.75 mg/kg)	Oral	8
Hoiland 2023	Nitric oxide	25 ± 5	22.9 ± 3 kg/m²	Both	Duplex ultrasound	End-tidal forcing to achieve SaO_2 of 75%	L-NMMA (5 mg/kg bolus dose followed by 5 µg/kg/min maintenance dose)	I.V.	11
Kellawan 2020	AA derivatives	28 ± 4	23 ± 1 kg/m²	Both	Transcranial doppler ultrasound	FiO_2 reduced from 21% to 11%	Indomethacin (100 mg)	Oral	9
Rocha 2020	K_{ATP} channels	24 ± 4	75.5 ± 6 kg	Male	Duplex ultrasound	FiO_2 reduced from 21% to 10%	Glibenclamide (5 mg)	Oral	10

Supplementary Table 5| Characteristics of selected animal studies.

7-NINA, 7-nitroindazole; 8-SPT, 8-(p-sulfophenyl)theophylline; 14,15-EEZE, 14,15 epoxyeicosa-5(Z)-enoic acid; AA, arachidonic acid; cAMP, cyclic adenosine monophosphate; CaO_2, arterial oxygen content; CBS, cystathionine β-synthase; CSE, cystathionine γ-lyase; FiO_2, fraction of inspired oxygen; HO-2, heme oxygenase-2; K_{ATP} channels, ATP-sensitive potassium channels; K_{ca} channels, calcium-sensitive potassium channels; L-NAME, N(G)-nitro-L-arginine methyl ester; L-NIO, N5-(1-iminoethyl)-L-ornithine; MS-PPOH, N-methylsulfonyl-6-(2-propargyloxyphenyl)henamide; PaO_2, arterial partial pressure of oxygen; SaO_2, arterial oxygen saturation; TTX, tetrodotoxin.

Publication	Pathway	Animals	Age	Weight	Sex	Technique	Method of induction of hypoxia	Intervention	Route of administration	Number of animals
Armstead 1995	Opiods	Piglets	1-5 days old	Not stated	Both	Pial artery diameter measurements	Inspired O_2 reduced to achieve PaO_2 of 25mmHg	β-funaltrexamine (10^{-8} M)	Applied on the brain surface	5
Armstead 1998	K_{Ca} channels	Piglets	1-5 days old	Not stated	Both	Pial artery diameter measurements	Inspired O_2 reduced to achieve PaO_2 of 25mmHg	Iberiotoxin (10^{-7} M)	Applied on the brain surface	5
Armstead 1999	Nitric oxide	Piglets	1-5 days old	Not stated	Both	Pial artery diameter measurements	Inspired O_2 reduced to achieve PaO_2 of 25mmHg	L-NNA (10^{-6} M)	Applied on the brain surface	8
Audibert 1991	Histamine	Dogs	Not stated	13.5± 0.6kg	Not stated	Radiolabeled microspheres	FiO_2 reduced from 21% to 10%	Famotidine (0.4 mg/kg), roxatidine (1 mg/kg)	I.V.	12
Audibert 1995	Nitric oxide	Dogs	Not stated	12.8± 0.5kg	Both	Radiolabeled microspheres	FiO_2 reduced from 21% to 10%	L-NAME (20 mg/kg)	I.V.	16
Audibert 1998	Histamine	Dogs	Not stated	14.5± 0.6kg	Not stated	Radiolabeled microspheres	FiO_2 reduced from 21% to 10%	D-chlorpheniramine (0.5 mg/kg)	I.V.	16
Ben Haim 2000	K_{Ca} channels and cAMP	Piglets	1-5 days old	Not stated	Both	Pial artery diameter measurements	Inspired O_2 reduced to achieve PaO_2 of 25mmHg	Iberiotoxin (10^{-7} M), Rp 8-Bromo cAMP (10^{-5}M)	Applied on the brain surface	14
Christie 2023	Nitric oxide	Rats	Young (specific age not stated)	100-150g	Not stated	Two-photon microscopy of cortical arteriole diameter	FiO_2 reduced from 21% to 10%	L-NAME (10 mg/kg)	I.V.	13
Coyle 1993	AA derivatives	Piglets	3-5 days old	1.63± 0.12kg	Not stated	Radiolabeled microspheres	FiO_2 reduced from 21% to 6-12%	Indomethacin (5 mg/kg)	I.V.	17
Coyle 1995	AA derivatives	Piglets	3-5 days old	1.67± 0.04kg	Not stated	Radiolabeled microspheres	FiO_2 reduced from 21% to 7-13%	Indomethacin (5 mg/kg)	I.V.	17
Kanu 2007	K_{Ca} channels	Piglets	1-3 days	Not stated	Not stated	Pial artery diameter measurements	FiO_2 reduced from 21% to 13%	Paxilline (4x10^{-5} M)	Applied on the brain surface	16
Kutzsche 2002	Nitric oxide	Piglets	1-3 days	1.5-2.5kg	Both	Laser doppler	FiO_2 reduced from 21% to 8%	L-NAME (5 mg/kg)	I.V.	16
Laudignon 1990	Adenosine	Piglets	1-3 days	1-1.5kg	Not stated	Radiolabeled microspheres	Inspired O_2 reduced to achieve PaO_2 of 40-60mmHg	8-phenyltheophylline (8 mg/kg)	I.V.	11
Liu 2015	AA derivatives, glutamate, adenosine, K_{ATP} Channels	Rats	Not stated	Not stated	Male	Laser doppler	FiO_2 reduced from 21% to 10%	14,15-EEZE (30 μM), MS-PPOH (20 μM), SCH58261 (1 μM), MRS1754 (1 μM), glibenclamide (10 μM), HET0016 (1 μM)	Applied on the brain surface	120
McPhee 1987	Adenosine	Piglets	1-5 days	1-1.7kg	Not stated	Radiolabeled microspheres	Inspired O_2 reduced to achieve PaO_2 of 20-30mmHg	Theophylline (10 mg/kg)	I.V.	16
Miekisiak 2008	Adenosine	Mice	Not stated	22-31g	Not stated	Laser doppler	FiO_2 reduced from 21% to 10%	ZM-241385 (1.0 mg/kg); genetic knockout of adenosine A2A receptors	I.V.	42
Morii 1987	Adenosine	Rats	Not stated	200-300g	Not stated	Radiolabeled microspheres	Inspired O_2 reduced to achieve PaO_2 of 30-40mmHg	Theophylline (0.2 μmol/g)	I.P.	29
Morikawa 2012	Hydrogen sulfide	Mice	Not stated	Not stated	Male	Two-photon microscopy of cortical arteriole diameter	FiO_2 reduced from 21% to 10%	Genetic knockout of HO-2, CBS, and CSE		31
Pellegrino 1993	Nitric oxide	Rats	Not stated	~400g	Male	Radiolabeled microspheres	FiO_2 reduced from 21% to 13%	L-NAME (3 mg/kg/min)	Applied on the brain surface	9
Pellegrino 1995	Nitric oxide, glutamate, sodium channels, adenosine	Rats	Not stated	400g	Male	Pial artery diameter measurements	FiO_2 reduced from 21% to 13%	8-SPT (10^{-5} M), MK-801 (10^{-5} M), TTX (10^{-6} M), L-NAME (10^{-3} M)	Applied on the brain surface	22
Shankar 1995	K_{ATP} channels	Piglets	1-5 days old	Not stated	Both	Pial artery diameter measurements	Inspired O_2 reduced to achieve PaO_2 of 36mmHg	Glibenclamide (10^{-6}M)	Applied on the brain surface	5
Simpson 1991	Adenosine	Rats	Not stated	Not stated	Not stated	Pial artery diameter measurements	FiO_2 reduced from 21% to 8%	Adenosine deaminase (0.5-2 U/ml)	Applied on the brain surface	6
Taguchi 1994	K_{ATP} channels	Rabbits	Not stated	2.3-2.7kg	Male	Pial artery diameter measurements	FiO_2 reduced from 21% to 8%	Glibenclamide (10^{-6} M)	Applied on the brain surface	8
Tomiyama 1999	K_{ATP} channels	Rats	Not stated	310-400g	Male	Indicator fractionation	Inspired O_2 reduced to achieve CaO_2 of 7.5ml O_2/dl	Glibenclamide (4×10^{-4} M)	Intracisternal	20
Wagerle 1983	Catecholamines	Sheep	Less than 2 weeks old	Not stated	Not stated	Radiolabeled microspheres	Inspired O_2 reduced to achieve PaO_2 of 25mmHg	Prazosin (0.5 mg/kg)	I.V.	42
Weiss 2001	Catecholamines	Rabbits	Not stated	1-2.4kg	Both	Radiolabeled microspheres	FiO_2 reduced from 21% to 10%	N-methyl chlorpromazine (4 mg/kg), phenoxybenzamine (2 mg/kg)	I.V.	21
Wilderman 1997	Nitric oxide, sodium channels	Piglets	1-5 days old	Not stated	Both	Pial artery diameter measurements	Inspired O_2 reduced to achieve PaO_2 of 25mmHg	7-NINA (10^{-6} M), TTX (10^{-6} M)	Applied on the brain surface	14
Wilderman 1998	Nitric oxide	Piglets	1-5 days old	Not stated	Both	Pial artery diameter measurements	Inspired O_2 reduced to achieve PaO_2 of 25mmHg	L-NIO (10^{-6} M),	Applied on the brain surface	12
